# Genetic typing of isolates of *Rickettsia typhi*

**DOI:** 10.1371/journal.pntd.0010354

**Published:** 2022-05-31

**Authors:** Cecilia Y. Kato, Ida H. Chung, Lauren K. Robinson, Marina E. Eremeeva, Gregory A. Dasch

**Affiliations:** Rickettsial Zoonoses Branch, Centers for Disease Control, Atlanta, Georgia, United States of America; Uniformed Services University of the Health Sciences, UNITED STATES

## Abstract

Murine typhus, which is caused by *Rickettsia typhi*, has a wide range of clinical manifestations. It has a low mortality rate but may result in meningoencephalitis and interstitial pneumonia in severe cases. Comparisons of complete genome sequences of *R*. *typhi* isolates from North Carolina, USA (Wilmington), Myanmar (B9991PP), and Thailand (TH1527) identified only 26 single nucleotide polymorphism (SNP) and 7 insertion-deletion (INDEL) sites in these highly syntenic genomes. Assays were developed to further define the distribution of these variant sites among 15 additional isolates of *R*. *typhi* with different histories from Asia, the USA, and Africa. Mismatch amplification mutation assays (MAMA) were validated for 22 SNP sites, while the 7 INDEL sites were analyzed directly on agarose gels. Six SNP types, 9 INDEL types, 11 total types were identified among these 18 isolates. Replicate DNA samples as well as comparisons of isolates with different passage and source histories gave consistent genetic typing profiles. Comparison of the SNP and INDEL markers to *R*. *typhi’s* nearest neighbor *Rickettsia prowazekii* demonstrated that the majority of the SNPs represent intra-species variation that arose post divergence of these two species while several INDEL sites also exhibited intraspecies variability among the *R*. *prowazekii* genomes that have been completely sequenced. The assays for the presence of these SNP and INDEL sites, particularly the latter, comprise a low technology gel method for consistently distinguishing *R*. *typhi and R*. *prowazekii* as well as for differentiating genetic types of *R*. *typhi*.

## Author summary

*Rickettsia typhi* is an obligately intracellular bacterium which is most commonly transmitted by rat fleas to humans and can cause the disease murine (endemic) typhus wherever both humans and infected rats are present. However, this agent is also present in a variety of other arthropod species associated with other vertebrate animals. Its closest relative is *Rickettsia prowazekii*, the etiologic agent of epidemic typhus (human louse-borne) and sylvatic typhus (associated with flying squirrels and its ectoparasites in the Eastern United States). Both classic louse-borne and flea-borne typhus rickettsiae can cause fatal infections and have the potential for use as biothreat agents as they can be grown in large quantities. We investigated the genetic differences that can be found in different stocks of *R*. *typhi*. Our work is important for two reasons. We have developed robust and sensitive methods for distinguishing isolates of *R*. *typhi* originating from different continents; these new assays require only simple instrumentation which is available in nearly all biology laboratories. Our results also provided new insights confirming the probable origin of *R*. *typhi* in Asia and its likely cosmopolitan spread by rats on ships around the world.

## Introduction

*Rickettsia typhi* is a gram-negative obligate intracellular bacterium transmitted most commonly by the Oriental rat flea, *Xenopsylla cheopis* [1]. Mammals such as *Rattus* spp. serve as the main reservoirs involved in the pathogen’s replication cycle [2]. The bacteria can be transmitted through flea saliva or shed in flea feces and infect the host through feeding or skin abrasions, the conjunctivae or by inhalation of aerosolized materials; however, the actual route of infection in many confirmed cases of murine typhus is unknown and many cases present with atypical syndromes [3–5]. Outbreaks of murine (endemic) typhus have been identified worldwide [4]. In the United States most cases of murine typhus occur in Hawaii, California and Texas [5–11]. Recent cases have also been reported from South America, Mexico, Europe, the Middle East, Africa, Asia, and Australia, [12–24] and travel-acquired cases are not uncommon [25–27]. Murine typhus manifests with fever including severe fever with thrombocytopenia, headache, vomiting, myalgia, malaise, and rash and generally has a low mortality rate [6,12–23], but may result in meningoencephalitis, interstitial pneumonia, shock, and respiratory failure in severe cases [4,22,24–25].

Murine typhus is most commonly suspected and confirmed serologically, primarily requiring the exclusion of epidemic typhus as a potential alternative etiology [27–29]. Although direct PCR [30], qPCR [31–33], LAMP [34], and RPA (recombinase polymerase amplification) [35] assays have been developed for detecting *R*. *typhi* in clinical samples, it is substantially easier to detect this agent in infected fleas because the quantity of organisms that are present can be much higher than in clinical or animal samples [9,11,36]. The simplest method for differentiating *R*. *typhi* from *R*. *prowazekii* isolates employs restriction fragment length polymorphism (RFLP) analysis of PCR amplicons [37,38]. Eremeeva et al. [37] were unable to differentiate any of the former USSR (Georgia, Azerbaijan) *R*. *typhi* isolates investigated from the reference US Wilmington strain by RFLP of their *glt*A, 17 kD protein antigen or 120 kD (*omp*B) genes even though they were readily distinguished from a diverse collection of *R*. *prowazekii* isolates by this method. While PCR-RFLP has been proven to be a simple and specific method for differentiating species of *Rickettsia*, nucleotide sequence analysis of PCR amplified target genes is also a rapid, sensitive and convenient technique for distinguishing these two species. Massung et al. [39] reported that isolates of *R*. *prowazekii* exhibited insertion deletion (INDEL) variation in their tandem repeats but could not differentiate isolates of *R*. *typhi* by this method. Consequently, the work reported here is the first which describes simple gel based PCR laboratory methods by which diverse isolates of *R*. *typhi* can be differentiated at a number of genetic loci.

Mismatch Amplification Mutation Assays (MAMA) have been used for genotyping applications to enhance discrimination between alleles [40–41]. These MAMA assays are PCR based tests that use a modified primer design to enhance detection of SNP differences and thus to discriminate between alleles. Insertion/deletion (INDEL) typing assays can also be used to detect the presence of genetic differences attributed to insertion or deletion events between species or isolates of *Rickettsia* using PCR [39,42]. DNA sequencing is not required for these assays, thus resulting in a rapid, simple, and inexpensive tool to differentiate between species and to characterize different isolates into specific groups or clades. Thus, one purpose of the present study was to develop this efficient and inexpensive method for *R*. *typhi* isolate discrimination for use in epidemiological investigations. By focusing on variant single nucleotide polymorphism (SNP) and insertion-deletion (INDEL) sites that were detected in the three complete genome sequences available during our laboratory study (we provided the purified isolate DNA for the genome sequencing of RtTH1527 and RtB9991PP), we succeeded in applying this low technology to group isolates of *R*. *typhi* into specific clades based on their genomic sequence differences. Some of these sites could also reliably differentiate *R*. *typhi* from isolates of *R*. *prowazekii* and add to the INDEL site assays previously described [39].

## Results

### Analysis of variant sites in three complete *R*. *typhi* genome sequences

Alignments of complete genome sequences of *R*. *typhi* isolates from North Carolina, USA (Wilmington), Myanmar (Burma) (B9991PP), and Thailand (TH1527) identified only 26 single nucleotide polymorphism (SNP) and 10 insertion-deletion (INDEL) sites in these highly syntenic genomes. Three INDEL sites due to single nucleotide deletions were not suitable for MAMA primer design and not studied further. Eighteen different isolates of *R*. *typhi* were available for analysis of the SNP and INDEL assays developed for evaluating these variant sites (Table 1). Three isolates (Bb, AZ331, and AZ332) were tested as replicate DNA samples (-a, -b), and two other isolates (Gear & HGear and FLA & HFLA) had different passage histories and origins so 23 total *R*. *typhi* DNAs were available for screening for variants with the assays.

**Table 1:** Isolates of *Rickettsia typhi* (Rt) and details of their origin.

Isolate*	Geographic Origin	Sample Source	Year	Source (Isolation Reference)#	DNA #
Rt B9991PPg	Myanmar (Burma)	*Rattus norvegicus*	1975	Univ. Maryland; C. L. Wisseman, Jr.	4
Rt B10056PP	Myanmar (Burma)	*Bandicota indica*	1975	Univ. Maryland; C. L. Wisseman, Jr.	14
Rt B72	Myanmar (Burma)	*Bandicota indica*	1975	Univ. Maryland; A.F. Azad	15
Rt Bb-a	Myanmar (Burma)	*Bandicota bengalensis*	1975	Univ. Maryland; A.F. Azad	19
Rt Bb-b	Myanmar (Burma)	*Bandicota bengalensis*	1975	Univ. Maryland; A.F. Azad	21
Rt TH1527g	Thailand	Human	1965	WRAIR; M. Bozeman	6
Rt TA837	Thailand	*Rattus exulans*	1963	WRAIR; M. Bozeman	9
Rt NA18PP	Pakistan	Rat	1970	Univ. Maryland; C. L. Wisseman, Jr.	11
Rt Museibov	Republic of Azerbaijan	Human	1949	Gamaleya Inst; M. E.Eremeeva (Kulagin) [37]	1
Rt Ger	Republic of Georgia	Human	1946	Gamaleya Inst; M. E.Eremeeva (Soliterman) [37]	2
Rt BII	Republic of Georgia	*Rattus norvegicus*	1946	Gamaleya Inst; M. E.Eremeeva (Soliterman)[37]	3
Rt AZ306	Ethiopia	*Rattus rattus*	1975–6	Univ. Maryland; A.F. Azad	13
Rt AZ331-a	Ethiopia	*Rattus rattus*	1975–6	Univ. Maryland; A.F. Azad	10
Rt AZ331-b	Ethiopia	*Rattus rattus*	1975–6	Univ. Maryland; A.F. Azad	23
Rt AZ332-a	Ethiopia	*Rattus rattus*	1975–6	Univ. Maryland; A.F. Azad	20
Rt AZ332-b	Ethiopia	*Rattus rattus*	1975–6	Univ. Maryland; A.F. Azad	22
Rt AZ357	Ethiopia	*Rattus rattus*	1975–6	Univ. Maryland; A.F. Azad	18
Rt Gear	South Africa	Human	1939	WRAIR; M. Bozeman (Gear) [43]	8
Rt GearH1268	South Africa	Human	1939	Harvard; J. Spielman (Gear) [43]	12
Rt Rat18	USA, California	Rat	1943	NIAID; ATCC (R. A. Ormsbee) [44]	7
Rt FLA	USA, Florida	*Rattus rattus*	1951	WRAIR; M. Bozeman (Rickard) [45]	5
Rt FLA H6590	USA, Florida	*Rattus rattus*	1951	Harvard; J. Spielman (Rickard) [45]	17
Rt WilmingtonPP	USA, North Carolina	Human	1928	NMRI; M.Bozeman/Dasch (Maxcy) [46]	16

*g, Complete genome sequence available; PP, Plaque Purified three times in primary chicken embryo fibroblasts; -a and–b are different passages and DNA preparations of the same isolate

#, Univ. Maryland: University of Maryland School of Medicine, Department of Microbiology and Immunology, Baltimore, MD; WRAIR: Walter Reed Army Institute of Research, Washington, DC; Gamaleya Inst.: the N.F. Gamaleya Scientific Research Institute of Epidemiology and Microbiology, Moscow, Russia; Harvard: Harvard School of Public Health, Cambridge, MA; NIAID: Rocky Mountain Laboratory, Hamilton, MT; NMRI: Naval Medical Research Institute, Bethesda, MD.

### Analysis of SNP sites with mismatch amplification mutation assays (MAMA) in 18 *R*. *typhi* isolates

**Fig 1:**
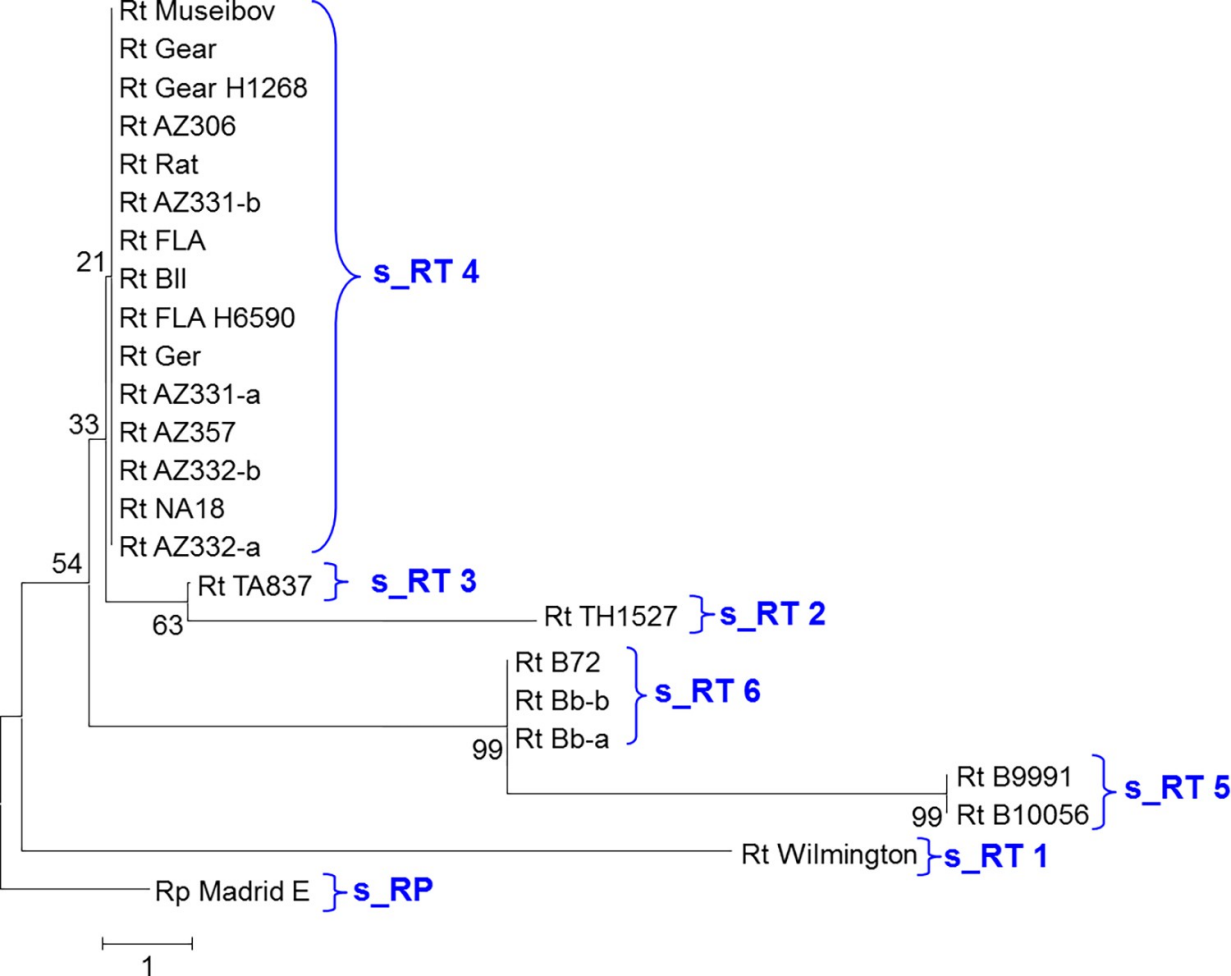
Neighbor joining tree analysis of the variant genetic sites found in isolates of *R*. *typhi*. Clades derived from 22 SNP sites.

**Fig 2:**
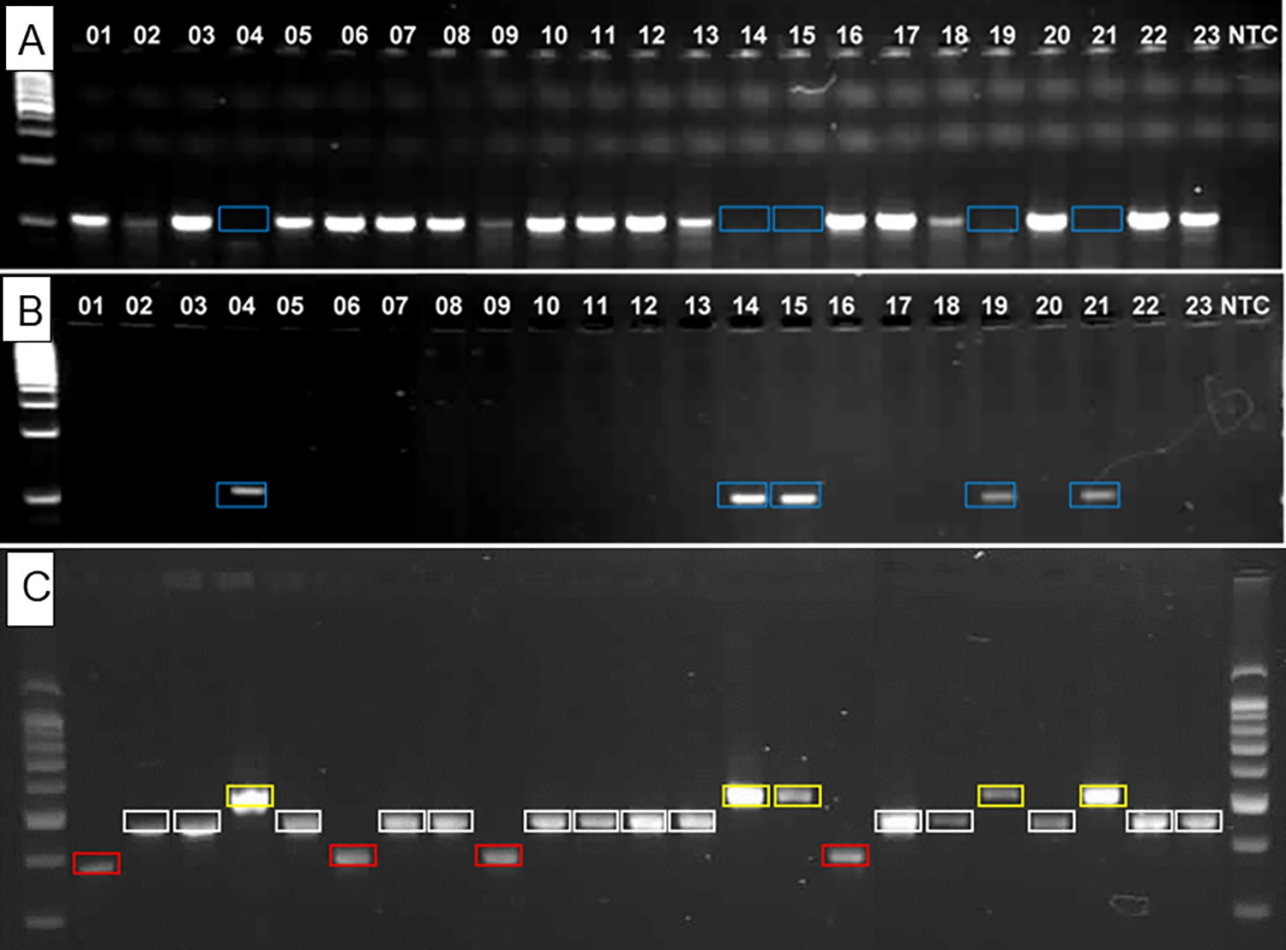
Representative SNP (MAMA) and INDEL assay results with 23 *R*. *typhi* DNAs. DNA numbers are listed in Table 1. The SNP assay for site 2_081 is shown with 2 selective primer sets for each polymorphism (A and B). Blue boxes in Panel A show no amplicons for this nucleotide type, and are the only amplicons produced in Panel B for the alternative nucleotide type. The INDEL assay (Panel C) for site i5_351 reveals 3 product sizes; yellow boxes 517 bp, while boxes 442 bp, and red boxes 367 bp.

MAMA assays were developed for 22 of the 26 SNP sites and used for discrimination of the variant alleles (S1 Table). The 23 *R*. *typhi* DNA preparations from 18 isolates (S2 Table and Fig 1) were each tested. The *R*. *typhi* Wilmington PP isolate was plaque purified while the genome sequence obtained for Wilmington was from a differently passaged uncloned stock of this isolate. All 22 SNP assays with these three pairs of isolate DNAs gave identical results (S2 Table). Six lineages of *R*. *typhi* and the *R*. *prowazekii* outgroup could be resolved with SNP assays (Fig 1). One representative gel result is shown for the SNP assay for site 2_081 (Fig 2A and 2B) and 4 other SNP assays are shown in S1 Fig. Wilmington, the most passaged isolate of *R*. *typhi* we examined, was readily separated from all the other isolates including the other two isolates from the USA (Fig 1). The other isolates from Africa, Eastern Europe and the USA (except Wilmington) could not be distinguished (Fig 1 and S2 Table). The strains from Asia exhibited the greatest diversity (Fig 1) as the two isolates from Thailand (TH1527, TA836) could be distinguished by 4 SNPs (1_058, 6_136–1, 16_453-I, 26_832) and both could also be differentiated from all other isolates by a single SNP (9_230) (S2 Table). The four isolates from Myanmar possessed 5 unique SNPs not found in other isolates of *R*. *typhi* (2_081, 12-348-I, 17_469, 20_554–1, 25_796); these Myanmar isolates could also be separated into two groups (Bb/B72 and B10056/B9991) with 5 additional SNPs (4_PG07, 7_140, 8_223, 11_290, 18_473) (S2 Table). Three SNP sites differentiated the *R*. *prowazekii* isolate sequences from the *R*. *typhi* alleles at the SNP locations (3 sites-T5, T12, T16 were not present in *R*. *prowazekii* and T20 was polymorphic site specific for *R*. *prowazekii*) (S3 Table).

### INDEL site analysis

INDEL sites in the 18 *R*. *typhi* isolate DNAs were also assessed by sizing the products produced by 7 PCR primer sets designed to flank the largest INDEL sites (Tables 2 and S4).

**Table 2:** Summary of INDEL site analyses (*sizes confirmed by sequencing, colored cells have variable size polymorphisms at that locus).

*R*. *typhi* Isolate	DNA	INDEL Sites						
		i1_032	i2_052	i3_162	i5_351	i7_600	i8_683–4	i10_825
Rt B9991PP	4	140*	1847*	144*	517	968*	1200	176*
Rt B10056PP	14	140	1847	144	517	968	1200	176
Rt B72	15	173	1397	144	517*	968	1200	159
Rt Bb-a	19	173	1397	144	517	968	1200	176
Rt Bb-b	21	173	1397	144	517	968	1200	176
Rt TH1527	6	173*	1397*	144*	367	968*	1200	159*
Rt TA837	9	173	1397	144	367	968	1200	159
Rt NA18PP	11	173	1397	144	442*	968	1200	159
Rt Museibov	1	173*	1397*	144	367	968	1200	159*
Rt Ger	2	173	1397	144	442*	968	1200	159
Rt BII	3	173	1397	144	442	968	1200	159
Rt AZ306	13	173	1097	144	442	968	1200	159
Rt AZ331-a	10	173	1397	144	442	968	1200	159
RT AZ331-b	23	173	1397	144	442	968	1200	159
Rt AZ332-a	20	173	1397	144	442	968	1200	159
Rt AZ332-b	22	173	1397	144	442	968	1200	159
Rt AZ357	18	173	1397	144	442*	968	1200	159
Rt Gear	8	173	1547	144	442	968	1200	159
Rt Gear H1268	12	173*	1547*	144	442	968	1200	159
Rt Rat 18	7	173	1397	144	442	884	1200	159
Rt FLA	5	173	1397	144	442*	968	1200	159
Rt FLA H6590	17	173	1397	144	442	968	1200	159
Rt WilmPP	16	173*	1397*	130*	367	884*	419	159*
RP_Madrid E	24	134	668	144	367	Repeat Region	1846	166

*sizes confirmed by sequencing, colored cells have variable size polymorphisms at that locus (sizes the same have same color)

**Fig 3:**
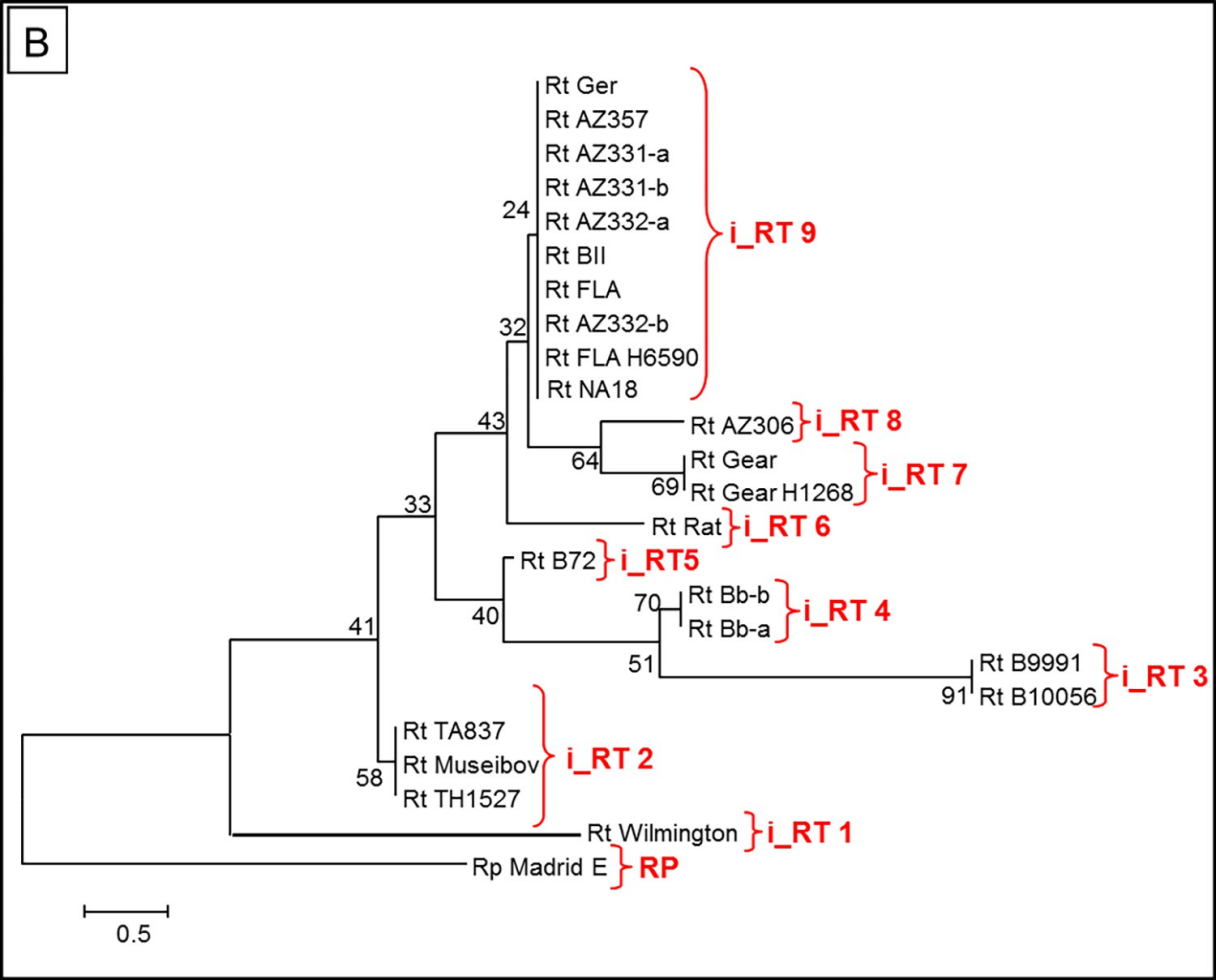
Neighbor joining tree analysis of the variant genetic sites found in isolates of *R*. *typhi*. Clades derived from 7 INDEL sites.

Representative INDEL typing results for the most informative site i-5_351 are shown in Fig 2C. Replicate DNAs (-a, -b) and isolates from different sources (Gear, H1268 Gear; FLA and FLA H6590) gave the same amplicon sizes (Fig 3). Nine *R*. *typhi* clades and the *R*. *prowazekii* Madrid E outgroup were resolved with the INDEL typing (Fig 3 and Table 2). One Rp INDEL site (i3_162) was predicted to be similar in size (144 bp) to the most common Rt INDEL, and a second Rp INDEL site (i5_351) was predicted to be similar in size (367 bp) to both the Rt Wilmington PP, the two Thailand isolates, and Rt Museibov (Table 2) and is shown in Fig 2. Sequence analyses of the five other INDEL sites contained unique polymorphisms in *R*. *prowazekii* relative to the *R*. *typhi* isolates (Table 2); however, the *R*. *typhi* primer sites contain mismatches to the *R*. *prowazekii* homologues (S2 Table) so a different set of primers as predicted in the Tables would be needed to amplify those variant Rt regions. The groupings for *R*. *typhi* were similar to those found by SNP typing (Fig 3) but with the following exceptions: Museibov grouped with the two Thai isolates (all three were identical) (clade i_RT2). All four Myanmar isolates had one unique INDEL amplicon (i5_351) (Table 2). The B72 differed by one INDEL site (i10_825) from the other three Myanmar isolates, B9991PP and B10056PP differed from B72 and Bb at two sites (i1_032, i2_052) (Table 2) and all four strains from Myanmar were divergent from all the other isolates (clades i_RT3, i-RT4 and i-RT5) (Fig 3). Finally, one USA strain (Rat 18) and two types of African strains (AZ306 and Gear/HGear) could be separated from each other and the rest of the large USA-African-Russian group of isolates (Clades i_RT6, i_RT7, iRT8) (Fig 3). Wilmington was again markedly different from all the other strains of *R*. *typhi* (clade i_RT1) and the PP strain DNA gave the identical INDEL profile as that predicted from the Wilmington genome sequence (Fig 3).

### Combined SNP and INDEL site analysis

**Fig 4:**
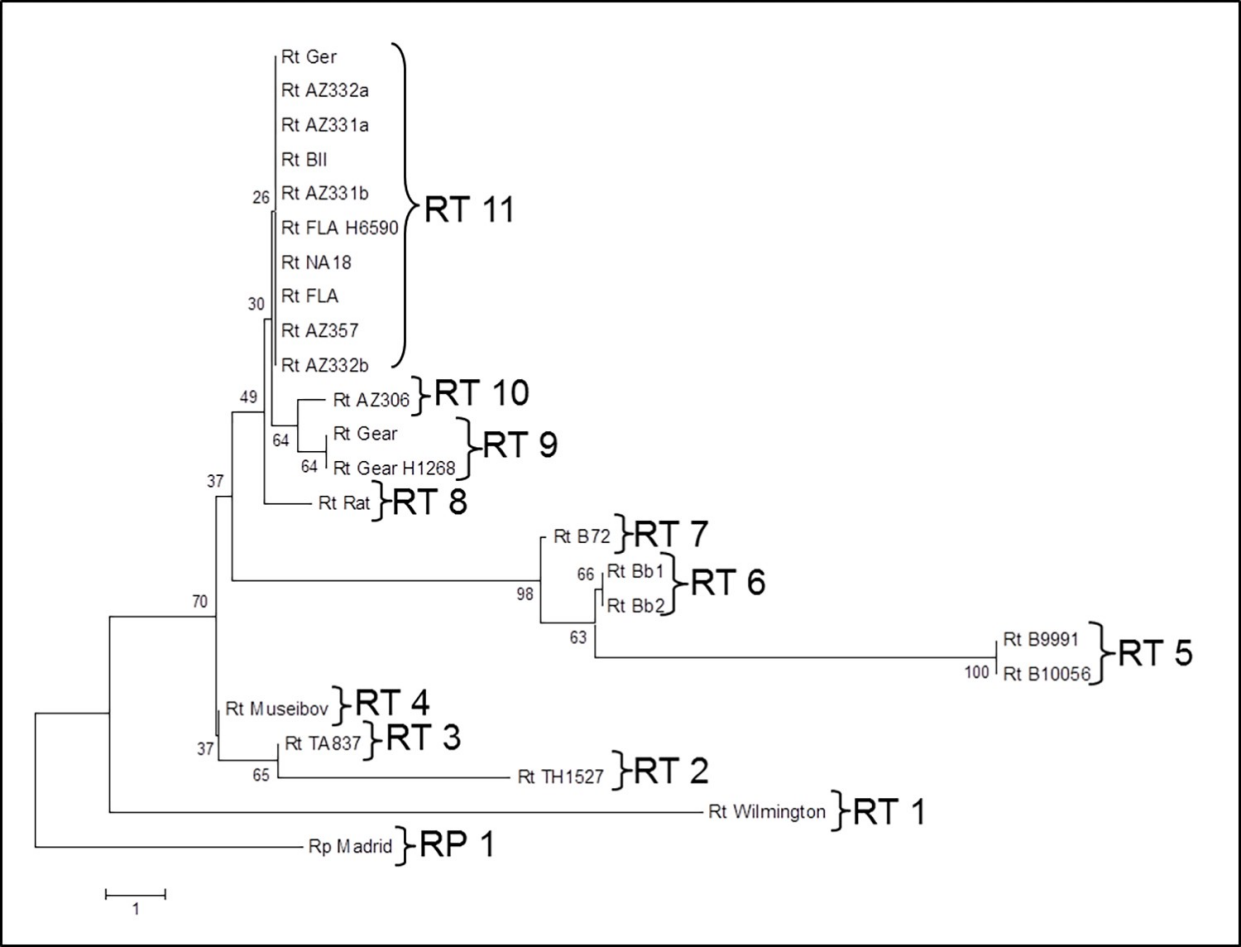
Neighbor joining tree analysis of the variant genetic sites found in isolates of *R*. *typhi*. Clades obtained by combining the SNP and INDEL site data. Bootstrap values are shown on the nodes.

The combined SNP and INDEL site data analysis is shown in Fig 4 and S5 Table. While the INDEL analysis exhibited greater resolution than that detected by SNP analysis, the geographic groupings were nearly the same as the individual SNP and INDEL clades (Figs 1 and 3), so that the primary benefit of using the combined data compared to INDEL typing is that longer branch lengths were obtained and the Museibov and two Thailand strains could be differentiated.

### Characteristics of unique sites in the genome sequence of *R*. *typhi* TM2540

MiSeq methodology was used to obtain an additional genome sequence for another isolate of *R*. *typhi* TM2540 from Thailand after our laboratory studies were completed (we do not have this isolate) [47]. In order to determine whether any additional variant sites were present in this isolate when compared to the Wilmington, TH1527, and B9991PP sequences, we performed a global alignment of the four sequences [48] (S6 Table). Thirty-four SNP or INDEL sites were unique to TM2540. Of the 21 sites with deletions, 15 were found in intergenic regions, 5 were in *sca*2 (no effect on reading frame), and 1 was in RT065_unk and caused a reading frame shift. One additional (147 bp) insert was in the repeat region of *sca*2 in the same region as the extra 450 bp repeat found in RtB9991PP. One 12 bp unique insert was in an intergenic region, a 16 bp insert caused a frameshift in RT0767_unk and a 552 bp deletion responsible for loss of coding sequences in both Rt0250_pbpB and Rt0251_xthA1. Of the 10 unique SNPs, 1 was in an intergenic region, 6 were non-synonymous substitutions in protein coding sequences and 3 caused synonymous substitutions.

For the 26 SNP sites originally identified among the three RtWilmington, RtB9991PP and RtTH1527 genomes, TM2540 shared 6 SNPs with RtWilmington and RtB9991PP, 11 SNPs with RtWilmington and RtTH1527, and 9 SNPS with RtB9991PP and RtTH1527 9. Among the 7 INDEL sites originally identified in the three RtWilmington, RtB9991PP and RtTH1527 genomes, TM2540 lacked the 14, 84, and 781 bp deletions found only in RtWilmington (i3_161–162, i7_600, and i8_683, respectively), the 33 bp deletion found in RtB999PP and RtB100056PP (i1_032), and the 17 and 150 bp inserts found only in RtB9991PP (i10_825–6 and i5_351, respectively). RtTM2540 also contained only a partial 147 bp fragment of the 450 bp insert found in RtB9991PP (i2_052).

## Discussion

Although *R*. *typhi* is a cosmopolitan species and has been spreading as a result of human activities that has distributed rats around the world on ships, carrying this agent with them [2], only a few genetic markers were found to exist among the isolates available to us even with complete genomic sequencing of isolates from opposite parts of the world (USA Wilmington, Thailand TH1527, and Myanmar B9991PP). The comparison of the whole genome sequences from the three different isolates resulted in only 26 SNPs and 10 INDEL differences (3 additional single bp deletions were not analyzed) among these three isolates. Strikingly, the highly passaged Wilmington strain genome sequence from a different laboratory [39] exhibited no differences from the sites we examined in the plaque purified Wilmington isolate we used. Seven SNPs and 2 INDEL sites we assayed were unique to Wilmington. This overabundance of unique sites in Wilmington suggests that they may have arisen during laboratory passage of these stocks and may be most useful in discriminating it as a potential contaminant in other isolates of *R*. *typhi*. Insufficient passage time exists between the cloned and uncloned stocks of this isolate to generate any differences in these sites although it is possible that other differences may be present elsewhere in the cloned genome. However, it also suggests that the original sequenced stock was clonal. B9991PP was a low passaged clonal stock, but could not be differentiated from the other cloned Myanmar B10056PP isolate with the assays developed here even though one was isolated from a bandicoot and the other from a rat. These observations suggest that these mutations can be stably maintained in nature. Moreover, the origin of mutations in *Rickettsia* by laboratory passage to attenuate *Rickettsia prowazekii* Madrid E and *R*. *rickettsii* Iowa is well documented [49,50].

In contrast to this independent experimental analysis of the variability of the sites with MAMA assays we identified from Wilmington, TH1727 and B9991PP genome sequences from Sanger (Wilmington) and or Sanger/454 sequencing, we were surprised at the number of additional sites reported in the TM2540 MiSeq data. The MinIon sequence reported for TM2540 contained an even larger number of apparent sequencing errors [47]. Until these unique sites in TM2540 are evaluated by an independent method (Sanger or MAMA), we are reluctant to consider them as being variant sites indicative of more genetic variation in this species. In particular, some of the sites would cause either deletions of protein genes we would expect to be functional and frame shifts in other protein genes which are conserved in the other three genomes. However, the pattern of variation is consistent with a strong relationship to the Asian isolates and with variability in isolates from Thailand (compare TA837 and TH1527) and the four Myanmar isolate profiles (S2 Table).

Wilmington, Gear, Museibov, Ger and TH1527, and TM2540 isolates were of human origin from cases of murine typhus. Since the human isolates all caused murine typhus, the genetic mutations in these strains did not attenuate their virulence sufficiently to prevent disease and suggests that it is likely all the strains of *R*. *typhi* circulating in nature are virulent for humans. We are unaware of consistent geographic differences in the pathogenicity of *R*. *typhi* for humans. The FLA (F2) strain from Florida and the Rat18 strain from California are very similar, differing in only one of the INDELs examined here, an INDEL (i7_600) in an 84 bp tandem repeat otherwise found only in *R*. *typhi* Wilmington. Changes in tandem repeat numbers are known to occur in *Rickettsia rickettsii* during laboratory passage [51] and between isolates of other species [39,42]. Six of the seven INDELs examined occurred in tandem repeats found by Tandem Repeat Finder [52] and five of them were in putative proteins.

The US isolates were genetically very close to many of the isolates from Africa, Eastern Europe and Pakistan. No differences were observed in the largest clade (RT 11) with 10 isolates from Georgia, Azerbaijan, USA, Africa, and Pakistan, as all targeted sites retained the most common SNP/INDEL pattern (Fig 3). The greater differences within isolates and relative to the African/US clade observed among *R*. *typhi* isolates from Myanmar and Thailand are consistent with the likely origin of this species in the Far East and the more ancient old world co-evolution of rats, oriental rat fleas, and *R*. *typhi* [2]. The isolates obtained from other parts of the world may have originated from a common lineage spread by humans, ships, and ship rats in more recent history although a few mutations among the African, US and European lineages were detected. Despite the wide range of habitats and isolate histories represented by our collection, it is very likely that other variant sites may be discovered by whole genome sequencing of additional isolates and by examination of other isolates from other rodent or vector hosts. It is possible that the differences found in isolates in the Myanmar and Thailand isolates suggests that immunological and ecological selective pressures arising from circulation in different vectors and reservoir hosts could give rise to novel genetic variants of *R*. *typhi*. However, notwithstanding this speculation, it is striking that so little apparent genetic variation was found to exist even between a US isolate with extensive passage in cell culture, bandicoot and rat isolates from Myanmar and human and rat isolates from Thailand or among the other isolates investigated here. It is therefore very likely that most of the wide spectrum of clinical differences observed for murine typhus among humans [1,4,6] are primarily due to human genetic, health, and nutritional factors and the infecting dose in those patients.

We believe that the primary utility of the assays described here will be in providing public health laboratories with new means to both differentiate *Rickettsia prowazekii* and *R*. *typhi* in clinical and environmental specimens obtained at sites were patients may have been infected. Because the assays were developed with genomic DNA, they will work best with DNA obtained from isolates or DNA amplified first with primers adjacent (flanking) to the MAMA and INSERT primer sites. Gel based PCR product size discrimination works best with significant size differences for INDEL assays but all of these assays work well with agarose gels. The best choice will depend largely on the geographic site from which the samples were obtained. That is the main consideration for choosing MAMA assays as well although it should be remembered that the same yield of amplicon will stain more intensely as its molecular weight increases and primer efficiency does vary between the assays. Alternative fluorescent dyes to ethidium bromide may also provide some increased assay sensitivity. These assays have not yet been validated by quantitative PCR approaches based on fluorescent dye yields but that may provide a possible alternative for their use for amplification of lower DNA copy samples.

## Materials and methods

### Isolate histories and preparation of DNA

The *R*. *typhi* isolates (Table 1) were obtained as yolk sac seeds and passaged one to six times in confluent Vero cells (strain C1008, green monkey kidney cells, obtained originally from the American Type Culture Collection, Manassas, VA) in RPMI 1640 supplemented with 2% fetal bovine serum, 2 mM L-glutamine and 5% tryptose phosphate broth. DNA was isolated from infected cell cultures and supernatants which had been harvested with 5 mm glass beads, pelleted at 12,000 rpm for 20 minutes in a Sorvall GSA rotor, and the pellet washed in PBS (1.0 ml per T150 flask). 0.25 ml of suspension was then lysed and extracted using QIAamp DNA Mini kit reagents (AL buffer plus proteinase K at 50°C overnight) and spin columns from Qiagen (Valencia, CA). DNA was eluted with 0.2 ml of AE buffer, diluted to 1.0 ml and stored at 4°C prior to analysis. Working DNA stocks (diluted 1:10 in water) were generally used for all analyses.

### Whole genome sequence analysis

SNP and INDEL sites were identified from alignments of the whole genome sequences of three *R*. *typhi* isolates, B9991CWPP = B9991PP (Myanmar) (GenBank NC017062.1), TH1527 (Thailand) (GenBank NC017066.1), and Wilmington (USA) genomes [53] using the MAVID alignment webserver [48]. All SNP and INDEL events are identified relative to the *R*. *typhi* strain Wilmington complete genome sequence coordinates (GenBank NC006142.1) [53] (S1 and S3 Tables). BLAST was used to identify the corresponding sites found in complete genome sequence of the *R*. *prowazekii* Madrid E isolate (NC_000963) as well as the genome sequences for the other 12 *R*. *prowazekii* isolates in GenBank (MadridE_NC_000963.1, MadridE_NMRC_NC_020992.1, Breinl_NC_020993.1, BuV67PP_NC_017056.1, Chernikova_NC_017049.1, Dachau_NC_017051.1, Katsinyian_NC_017050.1, Naples_NZ_CP014865.1, Rp22_NC_017560.1, GvF24_NC_017057.1, GvV257_NC017057.1, Cairo3_APMO01 [10 contigs], GvF12_APMN01 [20 contigs]). Subsequently when a sequence for *R*. *typhi* Laos isolate TM2540 became available [47], we prepared a MAVID alignment and used BLAST for obtaining the results shown in S6 Table.

### Single nucleotide polymorphism (SNP) assays

Suitable MAMA primers could be designed for 22 of the twenty-three SNP sites found among the three *R*. *typhi* isolates. Each SNP assay consists of two PCR reactions (2 primer sets) for each site (S1 Table). Each assay consists of two primer pairs: one standard primer and two nucleotide specific MAMA primers with the SNP of interest at the 3’ ultimate base of the primers. Because *Taq* polymerase is unable to extend the primer if there are two mismatch bases (3’-penultimate and 3’-ultimate base), MAMA primers were designed with a single mismatch created at the 3’ penultimate base for each SNP type, allowing the assay to differentiate the nucleotide of interest.

Five microliters of template DNA was amplified with each primer set (2 reactions per SNP site) using 12.5μl of the *Taq* PCR Master Mix (Qiagen, Valencia, CA) in a total volume of 25μl. PCR amplification was performed in 0.2 ml tubes using the Veriti Thermal Cycler (Applied Biosystems, Foster City, CA) using the following parameters: 94°C for 3 min, followed by 35 or 45 cycles of 94°C for 30 s, 50–60°C for 30 s, and 72°C for 30 s, and a final extension step of 72°C for 7 min (Table 2). PCR amplicons were resolved using gel electrophoresis with 3% ultrapure agarose (Invitrogen, Carsbad CA), 0.8X lithium borate buffer (Faster Better Media LLC, Hunt Valley MD), and GelStar Nucleic Acid Stain (Lonza, Rockland, ME) in 1X FBM loading buffer (Faster Better Media LLC, Hunt Valley MD). The gels were run for 30 to 60 min at 200 volts.

Assay conditions were optimized for each of the SNP target sites by adjusting primer concentrations, annealing temperatures, and the number of cycles. Optimal primer concentrations and cycling parameters were determined using the three reference *R*. *typhi* DNAs: B9991PP, TH1527, and Wilmington PP. A panel of 23 *R*. *typhi* DNA samples (18 isolates including 3 isolate technical replicates with different passage and extraction histories and 2 isolates with different laboratory origins) from humans and rodents were tested (Table 1).

### Insertion/Deletion assays

Primers flanking the INDEL sites were designed to produce the smallest amplicons possible consistent with amplification and resolution by agarose gel electrophoresis (predicted T_*m*_ >60°C) using Primer 3 (http://primer3.sourceforge.net/) [54] and are listed in S2 Table. PCR was performed with Qiagen Taq PCR Master Mix (Valencia, CA) and prepared at a final concentration of 1X master mix, 1 μM of each primer, and ~0.2 pg DNA in a 20 μl final volume. PCR was conducted in 96-well 9700 GeneAmp thermal cyclers (Applied Biosystems, Foster City, CA), 1 cycle at 95°C for 3 min followed by 35 cycles of 95°C for 30 s, 50°C for 30 s, and 72°C for 30 s; and a final cycle at 72°C for 7 min. PCR amplicon (with 1X FBM loading buffer, Faster Better Media LLC, Hunt Valley MD) separation was performed by agarose gel electrophoresis and visualized by UV imaging. Gels consisting of 3% ultrapure agarose were run as described above except 0.8X SB (sodium borate) buffer was used (Faster Better Media LLC, Hunt Valley MD) and gels electrophoresed for 90 to 570 min at 200 volts.

### Sequencing of PCR amplicons

Representative PCR amplicons of unexpected sizes (different from the three reference genome amplicons) were sequenced using the BigDye Terminator 3.0 Kit (Applied Biosystems) and DyeEx 2.0 spin kit (Qiagen) as specified by the manufacturer with a 3130xl Genetic Analyzer (Applied Biosystems). Amplified products were purified with Wizard PCR purification kits (Promega) directly or if secondary products were visible during analysis, the products were excised from agarose gels before purification. The electropherogram data was reviewed and the forward and reverse sequence information assembled with DNASTAR Lasergene 8.0.2. Sequences which were confirmed by sequencing were submitted to GenBank (#MT647569-MT647588, #MT655557-MT655561) and are indicated in Table 2.

### Genetic relationships

For each INDEL primer set, PCR amplicon sizes were expected to be B9991-like, TH1527-like, or Wilmington-like (designated as B, T, or U, respectively). If alternate amplicon sizes were detected, the differences were confirmed by sequencing and referenced in relation to the expected sizes using different letter designations. The out-group agent *R*. *prowazekii* Madrid E (NC_000963) was analyzed by BLAST and CLUSTALW alignments for each SNP and INDEL polymorphism location. The nucleotide data was summarized as character states and the alignment was generated with Molecular Evolutionary Genetics Analysis software (MEGA4) version 4.0 software [55]. Tree construction was performed using the neighbor joining method with the number of differences model.

## Supporting information

S1 Table SNP MAMA primer sites and sequences designed from comparison of complete genome sequences of *R*. *typhi* Wilmington (W), B9991PP (B), and TH1527 (TH) with primer sequences and Wilmington ORF location. Intergenic locations are indicated with a dash after the ORF closest to the chromosome origin. Variant sites in the MAMA primers are highlighted in red and the plus or minus indicates their directions within the intergenic regions relative to the ORFs or pseudogene (e. g., 4_PG07).
(XLS)

S2 Table Summary of SNP sites identified in DNAs from 18 isolates of *R*. *typhi* SNPs are identified by color in each locus column. The name of the respective open reading frame (ORF) or intergenic region locus (IGR) is indicated at the bottom of each column.
(XLS)

S3 Table Comparison of MAMA primers to 13 *R*. *prowazekii* genome sequences from NCBI (listed in Materials and Methods). (XLS)

S4 Table INDEL sites and sequences designed from comparison of complete genome sequences of *R*. *typhi* Wilmington (W), B9991 PP (B), and TH1527 (TH) with primer sequences and Wilmington ORF location. Intergenic locations are indicated with a dash after the ORF closest to the chromosome origin. Variant sites in the MAMA primers are highlighted in red and the plus or minus indicates their directions within the intergenic regions relative to the ORFs.
(XLS)

S5 Table Summary table of genetic variation by clade identified from SNPs, INDELs, and the combined data in 18 isolates of *R*. *typhi*. (XLS)

S6 Table Comparison of variant sites among four *R*. *typhi* genomes available at NCBI (Wilmington, TH1527, B9991PP, and TM2450). (XLS)

S1 Fig Four representative SNP (MAMA) results (sites 6_136–1, 7_140, 9_230, 24_764) with 23 *R*. *typhi* DNAs. DNA numbers are listed in Table 1.
(XLSX)
